# CytoSorb in patients with coronavirus disease 2019: A rapid evidence review and meta-analysis

**DOI:** 10.3389/fimmu.2023.1067214

**Published:** 2023-01-31

**Authors:** Shilin Wei, Yanchun Zhang, Kerong Zhai, Jian Li, Mingming Li, Jianbao Yang, Rongzhi Zhang, Yongnan Li, Zhenzhen Li

**Affiliations:** ^1^ Department of Thoracic Surgery, Lanzhou University Second Hospital, Lanzhou University, Lanzhou, China; ^2^ Laboratory of Extracorporeal Life Support, Lanzhou University Second Hospital, Lanzhou University, Lanzhou, China; ^3^ Department of Cardiac Surgery, Lanzhou University Second Hospital, Lanzhou University, Lanzhou, China; ^4^ Department of Anesthesiology, Lanzhou University Second Hospital, Lanzhou University, Lanzhou, China; ^5^ Department of Extracorporeal Circulation, Lanzhou University Second Hospital, Lanzhou University, Lanzhou, China

**Keywords:** CytoSorb, coronavirus disease 2019, cytokines, cytokine hemadsorption, meta-analysis

## Abstract

**Background:**

After its approval by the European Union in 2011, CytoSorb therapy has been applied to control cytokine storm and lower the increased levels of cytokines and other inflammatory mediators in blood. However, the efficiency of this CytoSorb treatment in patients with coronavirus disease (COVID-19) still remains unclear. To elucidate the Cytosorb efficiency, we conducted a systematic review and single-arm proportion meta-analysis to combine all evidence available in the published literature to date, so that this comprehensive knowledge can guide clinical decision-making and future research.

**Methods:**

The literature published within the period 1 December 2019 to 31 December 2021 and stored in the Cochrane Library, Embase, PubMed, and International Clinical Trials Registry Platform (ICTRP) was searched for all relevant studies including the cases where COVID-19 patients were treated with CytoSorb. We performed random-effects meta-analyses by R software (3.6.1) and used the Joanna Briggs Institute checklist to assess the risk of bias. Both categorical and continuous variables were presented with 95% confidence intervals (CIs) as pooled proportions for categorical variables and pooled means for continuous outcomes.

**Results:**

We included 14 studies with 241 COVID-19 patients treated with CytoSorb hemadsorption. Our findings reveal that for COVID-19 patients receiving CytoSorb treatment, the combined in-hospital mortality was 42.1% (95% CI 29.5–54.6%, I^2^ = 74%). The pooled incidence of adjunctive extracorporeal membrane oxygenation (ECMO) support was 73.2%. Both the C-reactive protein (CRP) and interleukin-6 (IL-6) levels decreased after CytoSorb treatment. The pooled mean of the CRP level decreased from 147.55 (95% CI 91.14–203.96) to 92.36 mg/L (95% CI 46.74–137.98), while that of IL-6 decreased from 339.49 (95% CI 164.35–514.63) to 168.83 pg/mL (95% CI 82.22–255.45).

**Conclusions:**

The majority of the COVID-19 patients treated with CytoSorb received ECMO support. In-hospital mortality was 42.1% for the COVID-19 patients who had CytoSorb treatment. Both CRP and IL-6 levels decreased after Cytosorb treatment.

## Introduction

After a new disease caused by the novel coronavirus broke out in Wuhan, China, millions of confirmed cases of coronavirus disease 2019 (COVID-19), were reported. The disease led to numerous deaths (1). Because of its high mortality rate, COVID-19-induced acute respiratory syndrome has put considerable strain on healthcare systems (2). Although it has previously been reported that during outbreaks of emerging infections, acute respiratory distress syndrome (ARDS) could be treated with extracorporeal membrane oxygenation (ECMO) (3, 4), the clinical outcome varies for COVID-19 patients with ECMO support (5–7). Hence, the same strategy cannot be adopted for COVID-19 cases.

ECMO support could provide stable hemodynamics and oxygen saturation, which is very recommended for patients with severe ARDS (8). However, COVID-19 is additionally associated with the excessive release of pro-inflammatory cytokines, such as granulocyte-macrophage colony-stimulating factor (GM-CSF), interleukin-6 (IL-6), interleukin-1 (IL-1), monocyte chemoattractant protein-1 (MCP-1), and tumor necrosis factor (TNF). This excessive release of the pro-inflammatory cytokines initiates an extreme inflammatory process and results in a cytokine storm (9). COVID-19 patients in the intensive care unit (ICU) had levels of interleukin-10 (IL-10), interleukin-2 (IL-2), interleukin-7 (IL-7), and TNF-α that were considerably higher than those of the non-ICU patients (10). This observation suggests that cytokines may be crucial in the pathophysiology of COVID-19 (11, 12). As an adjuvant therapy for systemic inflammation, CytoSorb therapy is an adsorptive blood purification procedure that attempts to lower the increased levels of cytokines and other inflammatory mediators in the blood and regulate the cytokine storm (13). Following its approval by the European Union (EU) in 2011, CytoSorb has been used to treat over 130,000 patients globally. It has been mainly used to treat systemic hyperinflammation and refractory shock (14). By controlling the cytokine storm, CytoSorb might be a promising choice for COVID-19 patients.

Owing to the complex pathophysiological environment of COVID-19, involving multiple mediators and highly redundant, overlapping feedback mechanisms, cytokine adsorption therapy may benefit from removing a wide range of inflammatory substances and other cytokines, at least in theory (15). Nevertheless, very few studies reported the outcomes after CytoSorb treatment in COVID-19 patients. Reaching a credible conclusion with such a small number of studies is difficult, and hence, we need some strong evidence to guide the choices for clinical treatment. The purpose of this study is to combine all the evidence in the published literature related to cytokine adsorption therapy, especially the use of the CytoSorb adsorption column for COVID-19 patients, and provide credible guidance for clinical practice.

## Methods

### Search strategy

The Preferred Reporting Items for Systematic Reviews and Meta-Analyses (PRISMA) Statement was followed when conducting this study (16). Using the Cochrane Library, PubMed, Embase, and the International Clinical Trials Registry Platform (ICTRP), we performed a comprehensive online search from 1 December 2019 to 31 December 2021, using the following: “COVID-19”, “2019 novel coronavirus”, “coronavirus disease 2019”, “2019-nCoV”, “SARS-CoV-2”, “severe acute respiratory syndrome coronavirus 2”, “CytoSorb”, “CytoSorb cartridge”, “CytoSorb hemadsorption”, “cytokine hemadsorption”, “cytokine adsorber”, and “cytokine adsorption” as MeSH and EMTREE keywords. To find and include relevant studies, we evaluated all related studies and their citations.

### Study eligibility

We identified and included all relevant studies published regarding CytoSorb treatment in adult patients with COVID-19. We excluded any animal studies, review articles, case reports of a single patient, or pediatric studies (< 18 years). The latest publication was used when multiple articles relating to a single study had been published, to avoid the possible overlapping of patients. Literature searches, study eligibility assessments, and data extraction was conducted by two independent investigators (S.L.W and Y.C.Z). By consensus and consultation with experienced reviewers (Z.Z.L and Y.N.L), discrepancies were resolved.

### Data collection

Two independent investigators (S.L.W and Y.C.Z) abstracted data using a predefined standardized protocol and tool. WebPlotDigitizer 4.5 Software (https://automeris.io/WebPlotDigitizer) was used to extract data from graphics. Two experienced reviewers (Z.Z.L and Y.N.L) consulted with each other and settled any disagreements. The data collection included study characteristics (first author, publication year, country); patient demographics (numbers of patients, age, body mass index [BMI], the proportion of male patients, comorbidities including hypertension and diabetes); pre-CytoSorb characteristics (Acute Physiology and Chronic Health Evaluation II [APCHE II] score, Sequential Organ Failure Assessment [SOFA] score, partial pressure of arterial oxygen to fraction of inspired oxygen ratio [PaO2/FiO2], serum C-reactive protein [CRP] and interleukin-6 [IL-6] levels); post-CytoSorb characteristics (serum CRP and IL-6 levels); mortality (both in-hospital and substitution of the nearest common mortality time point); and length of stay in ICU.

### Assessment of risk of bias

Joanna Briggs Institute (JBI) checklists were used to assess the study quality of case series and cohort studies. Egger’s test was employed to estimate publication bias. I^2^ statistics, Chi-squared tests, and visual inspections of forest plots were used to assess statistical heterogeneity.

### Outcomes of interest

In-hospital mortality was the primary outcome of interest. The secondary outcomes were serum IL-6 and CRP levels before and after CytoSorb treatment, length of ICU stay, duration of mechanical ventilation, adjunctive ECMO therapy, and mortality in patients with or without ECMO.

### Statistical analysis

The continuous variables were presented as mean (± standard deviation) or median (interquartile range). We used the formulas available in the literature to convert the median (interquartile range) into a mean (± standard deviation) (17, 18). We reported the categorical variables as numbers and percentages (%). To calculate summary effects, we used R 3.6.1 software. The summary effects were presented with 95% confidence intervals (CIs). Both odds ratios (OR) and mean differences (MD) were used as summary statistics for dichotomous data and continuous data, respectively. The heterogeneity of the studies was assessed by the I^2^. A random effects model was applied if the I^2^ value was greater than 50%, otherwise, a fixed effects model was used.

## Results

### Study identification

**Figure 1:**
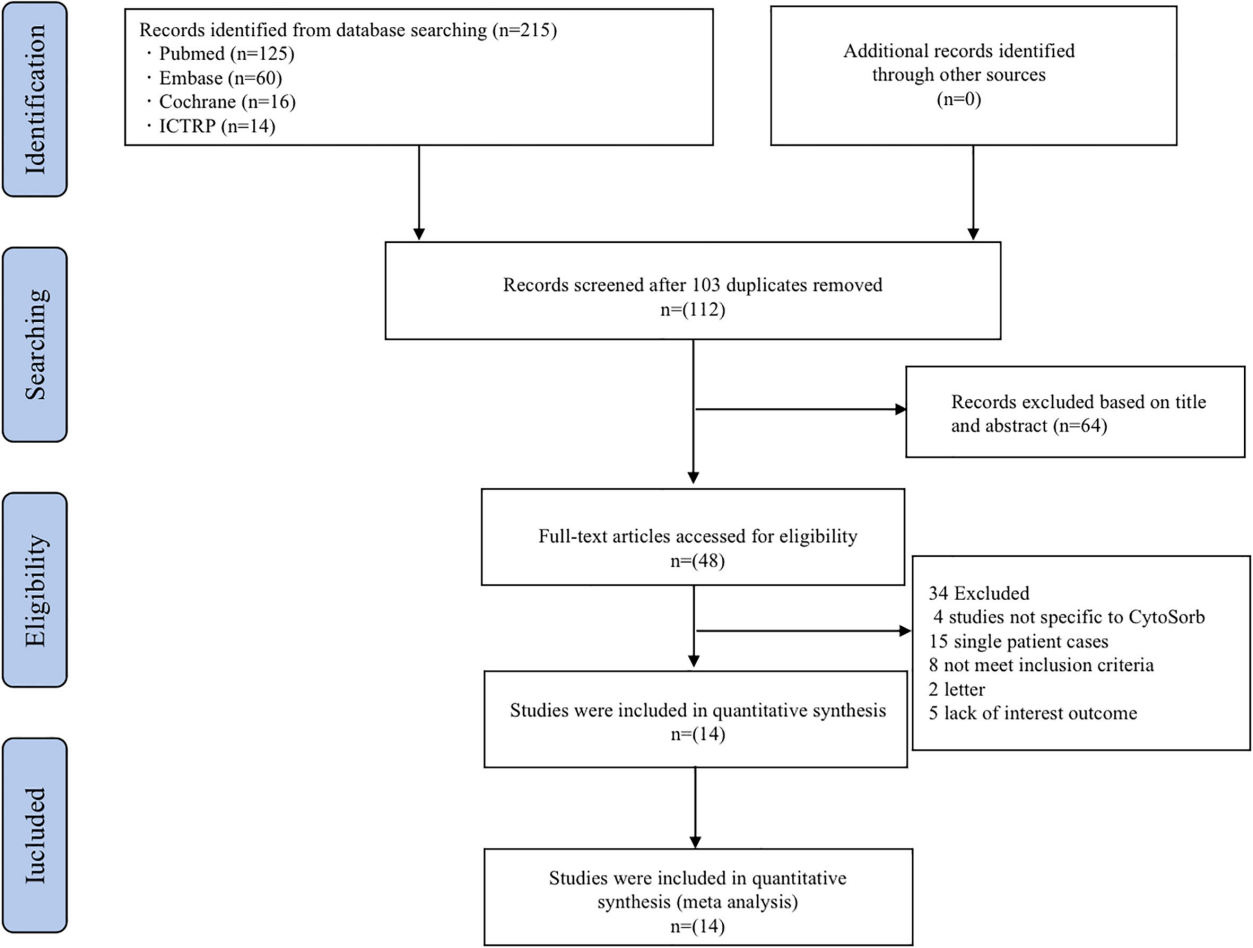
Flow diagram of the study selection process. ICTRP, the International Clinical Trials Registry Platform.

We identified 215 potentially relevant studies, removed 103 duplicates, and excluded 64 studies after carefully reviewing the titles and abstracts. A full-text review of the remaining 34 studies led to their exclusion, details can be found in **Figure 1**. Following a strict appraisal, 14 studies were selected, including 10 case series and 4 cohort studies. These studies included a total of 241 COVID-19 patients treated with CytoSorb hemadsorption (19–32).

### Baseline characteristics of the included studies

241 patients from 11 different countries were enrolled in the study, characteristics were summarized in **Table 1**. All of the included studies dealt with patients who were infected with the 2019 novel coronavirus. Sex was reported in 13 studies and 75.52% of patients were male. In 4 studies, all included patients were male. Age was reported in 13 studies including 178 patients, the pooled mean age was 54.29 (95% CI 50.54–58.05). Notably, hypertension and diabetes were the most common comorbidities in this population. The pooled incidence of hypertension is 48.2% (95% CI 33–63.4%) and that of diabetes is 31% (95% CI 22.1–39.9%).

**Table 1:** Demographics of the included studies.

First author	Country	Sample size	Male (n)	Age	P/F ratio	Comorbidity	CytoSorb management (Duration and flow rate)
Alharthy	Arabia	50	39	49.64 ± 8.90	117.46 ± 36.92	Hypertension:25 Diabetes:14	Patients had 2 ± 1 sessions; Flow rate: 180 ± 20 ml/min
Damiani	Italy	11	11	59.21 ± 12.55	108.51 ± 38.17	Hypertension:7 Diabetes:3	Two patients with one 24-hour cycle, the remaining 9 were 48 hours; Flow rate: 100–150ml/min
Lebreton	France	11	NR	NR	NR	NR	Each patients had 48 hours on CytoSorb
Lewis	USA	8	7	41.13 ± 11.62	90.1 ± 45.57	NR	A total treatment duration of 72 hours
Mehta	India	3	2	54.33 ± 12.01	108.1 ± 51.5	Hypertension:3 Diabetes:2	24 hours duration; Flow rate: 150–700mL/min
Nassiri	Iran	26	20	53.7 ± 16.13	177.54 ± 41.49	Hypertension:16 Diabetes:14	Number of CytoSorb cartridges used 2 [1-3]; duration 35 [18-48] h; Flow rate: 200-250mL/min
Paisey	UK	15	12	50.64 ± 5.73	NR	Hypertension:1 Diabetes:5	Cartridge was replaced q12h in the first 24 h, q24h for the subsequent 48 h; Flow rate >150ml/min
Peng	China	10	8	67.7 ± 10.41	142.02 ± 119.54	Hypertension:5 Diabetes:2	CytoSorb duration 47 [12-92] h; Flow rate: 150ml/min
Pieri	Italy	15	15	55 ± 14	112 ± 36	NR	Each patient received an average of three cycles. The mean duration time was 17 h 21 min; Flow rate: 150-200mL/min
Rampino	Italy	5	5	57.8 ± 3.39	259.8 ± 52.32	Hypertension:2 Diabetes:0	4 h sessions in 2 consecutive days; Flow rate: 100-120mL/min
Rodeia	Portugal	5	5	45 ± 17.9	79.2 ± 23.19	Hypertension:2 Diabetes:1	Two sequential CytoSorb cartridges each for 24 h
Supady	Germany	17	12	62.54 ± 14.15	61.18 ± 19.56	Hypertension:9 Diabetes:5	cartridges were replaced every 24 h for a total treatment duration of 72h; Flow rate: 100-700mL/min
Virág	Hungary	13	12	57 ± 11	127.8 ± 58.48	Hypertension:9 Diabetes:6	96 hours; Flow rate: 100-250mL/min
Song	USA	52	34	48.71 ± 9.15	NR	Hypertension:21 Diabetes:17	Duration of CytoSorb therapy: 79.4 ± 27.72 hours

### Assessment of study quality

To appraise study quality for cohort studies and case series, we used the Joanna Briggs Institute (JBI) checklist. The results showed that all case series studies had scores above 8/10 and cohort studies had scores above 9/11 (**Supplementary Table 1**). This indicated that high quality was strictly maintained for our study.

### Pre−CytoSorb variables

BMI was reported in 8 studies and the pooled mean BMI was found to be 29.54 (95% CI 27.36–31.72). 11 studies reported PaO2/FiO2 before CytoSorb initiation. The pooled mean PaO2/FiO2 was 123.81 (95% CI 92.46–155.16). Pre-CytoSorb SOFA score before CytoSorb initiation was reported in 9 studies with a pooled mean SOFA score of 9.6 (95% CI 6.81–12.4). Pre-CytoSorb APACHE2 score before CytoSorb initiation was reported in 4 studies with a pooled mean APACHE2 score of 22.75 (95% CI 16.63–28.87). Serum CRP before CytoSorb initiation was reported in 10 studies with a pooled mean CRP of 147.55 mg/L (95% CI 91.14–203.96). Serum IL-6 before CytoSorb initiation was reported in 10 studies with a pooled mean IL-6 of 339.49 pg/mL (95% CI 164.35–514.63).

### Primary outcomes

#### In hospital mortality

**Figure 2:**
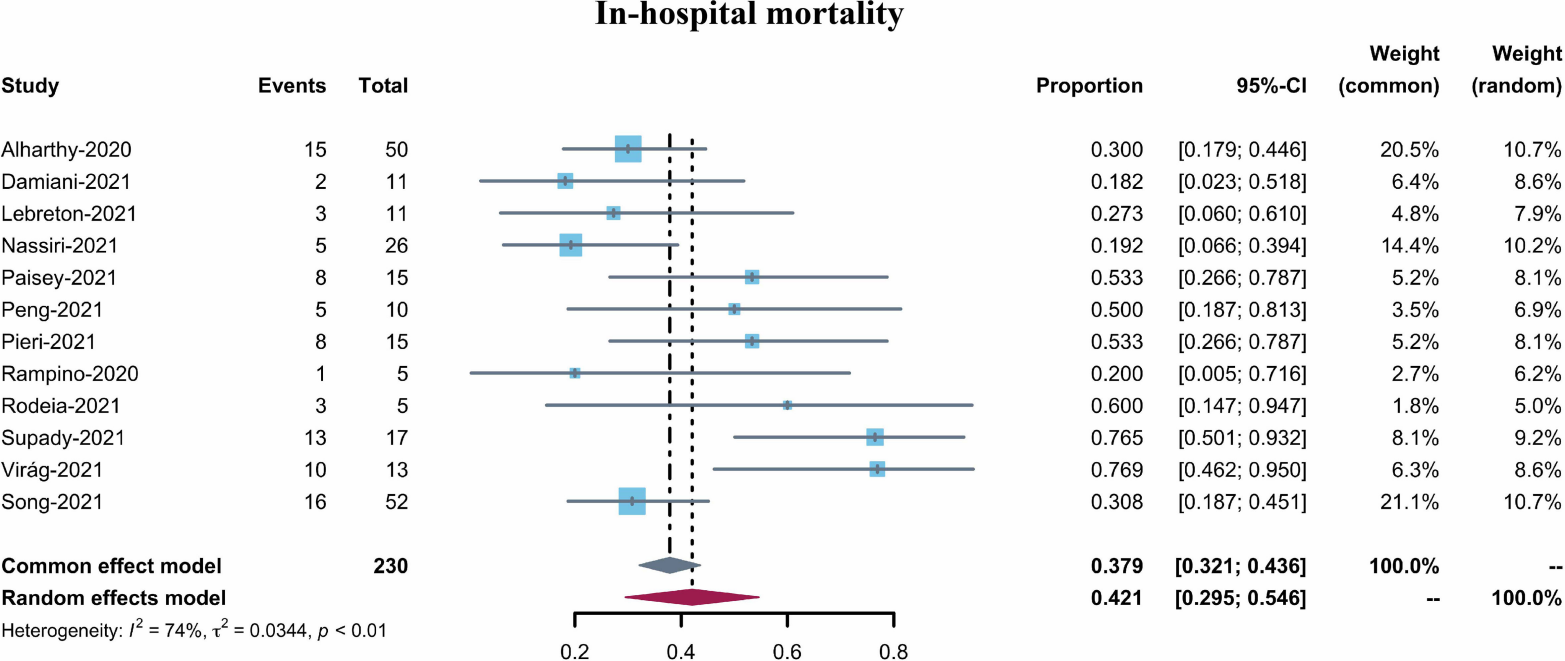
Forest plot of in-hospital mortality for COVID-19 patients with CytoSorb treatment. CI: confidence interval.

The pooled in-hospital mortality of COVID-19 patients receiving Cytosorb (12 studies, 230 patients) was 42.1% (95% CI 29.5–54.6%, I^2^ = 74%) (**Figure 2**) However, the in-hospital mortality reported varied from 18.2–76.9%. Visual inspection of the funnel plot indicated a low risk of publication bias (**Supplementary Figure 1**). In addition, Egger’s tests confirmed the absence of any overt risk of publication bias in the studies included (Pegger = 0.2194) (**Supplementary Figure 2**). A sensitive analysis (using the single-study-removed method) was also performed, which demonstrated decent stability in the primary outcome of in-hospital mortality (**Supplementary Figure 3**).

### Secondary outcomes

#### Serum IL-6 and CRP

**Figure 3:**
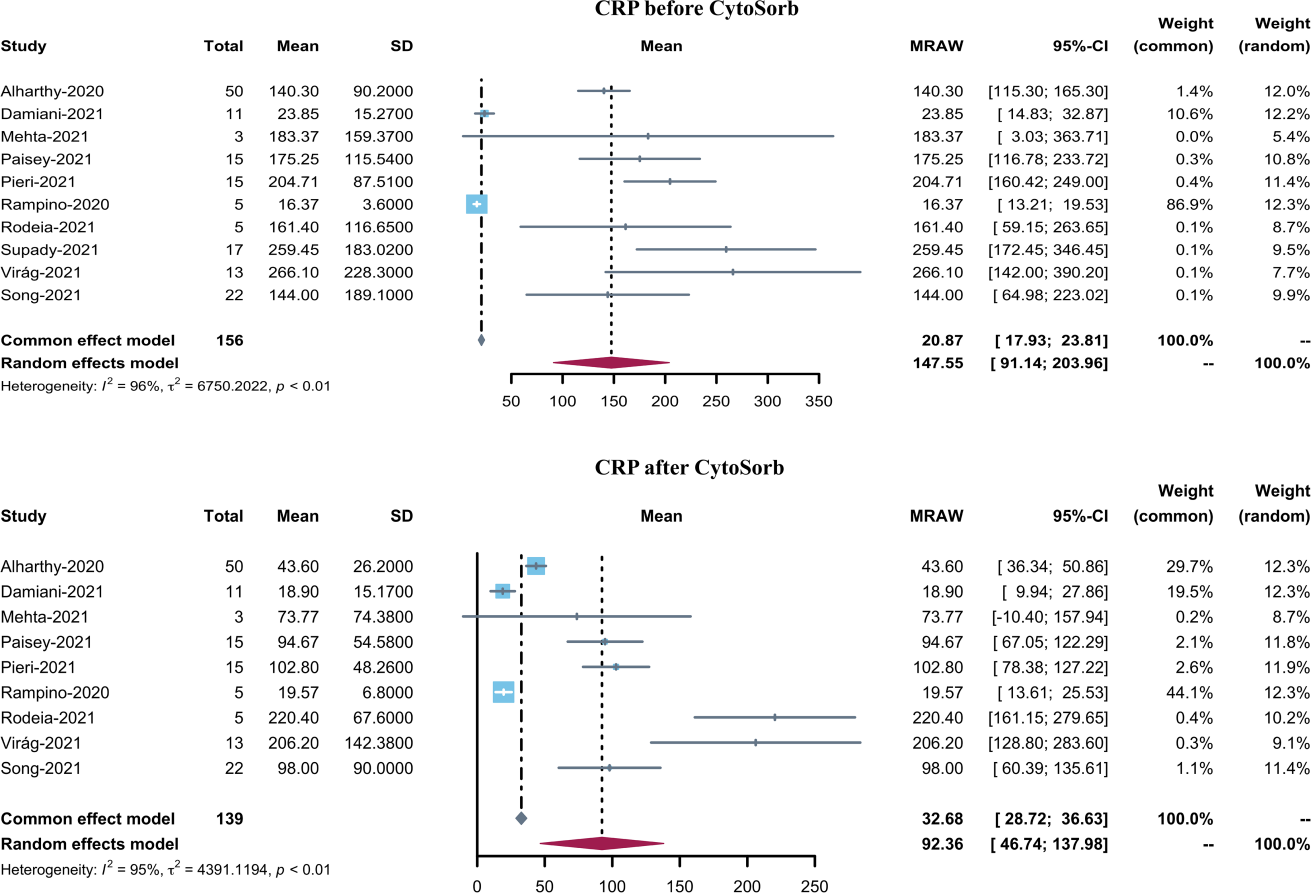
Forest plot of serum CRP before and after CytoSorb treatment for COVID-19 patients. CI: confidence interval; SD: standard deviation; CRP: C-reactive protein.

**Figure 4:**
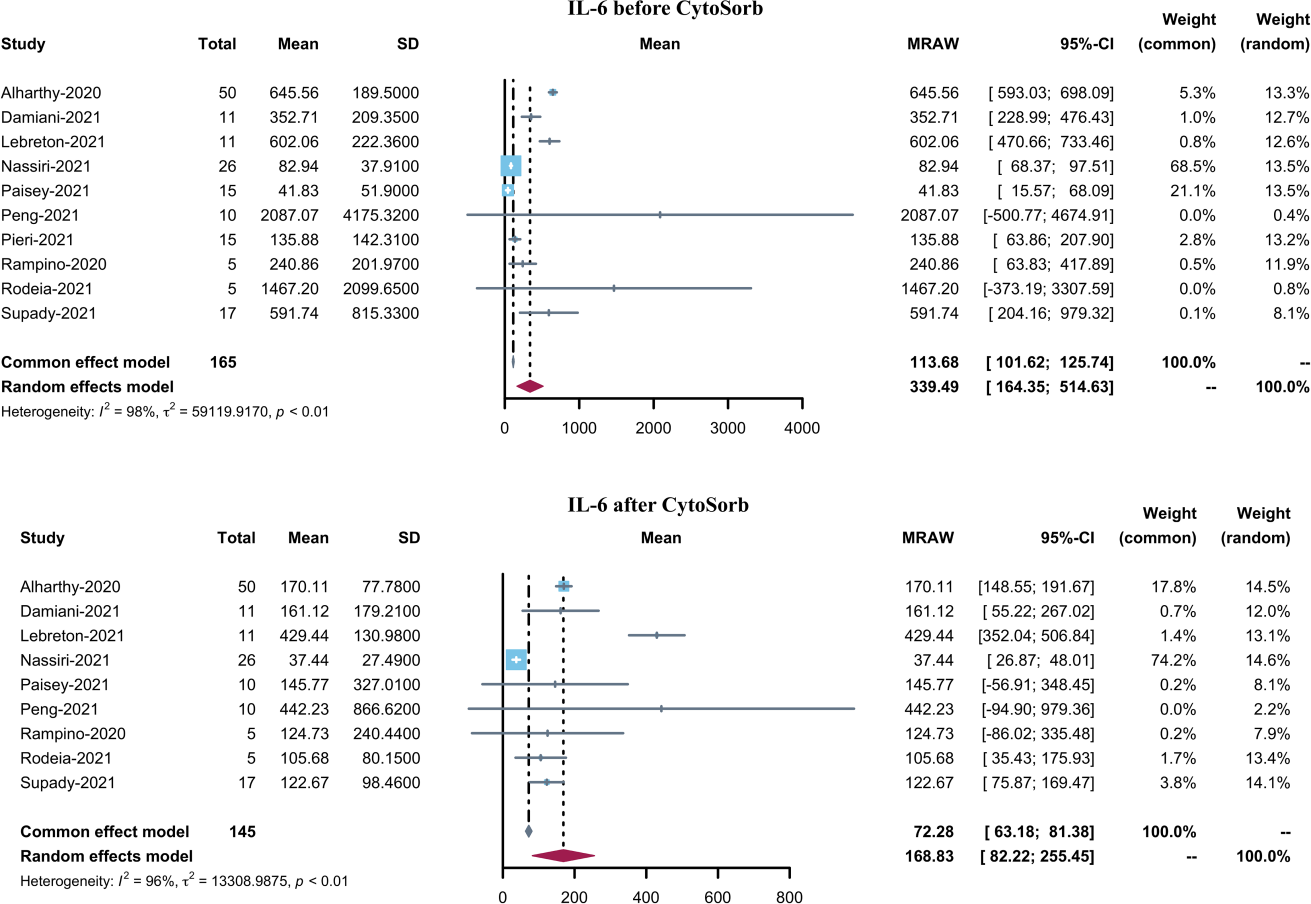
Forest plot of serum IL-6 before and after CytoSorb treatment for COVID-19 patients. CI: confidence interval; SD: standard deviation; IL-6: interleukin-6.

Ten studies reported serum IL-6 levels in COVID-19 patients after CytoSorb hemadsorption treatment. The mean serum IL-6 level varied from 37.44 to 442.23 pg/mL, with a pooled mean of 168.83 pg/mL (95% CI 82.22–255.45, I^2^ = 96%) (**Figure 3**). It is to be noted that the pooled mean IL-6 level before CytoSorb initiation was 339.49 pg/mL (95% CI 164.35–514.63). Nine studies reported post-CytoSorb hemadsorption treatment serum CRP in COVID-19 patients. The mean serum CRP varied from 18.9 to 220.40, with a pooled mean of 92.36 (95% CI 46.74–137.98) (**Figure 4**).

### Duration of mechanical ventilation, duration of ICU stay, and incidence of ECMO support

Four studies reported the duration of mechanical ventilation (MV) after CytoSorb hemadsorption treatment with a pooled mean of 14.37 days (95% CI 7.96–20.78, I^2^ = 94%). Six studies reported length of stay in the ICU with a pooled mean of 19.74 days (95% CI 12.92–26.56, I^2^ = 96%). Except for CytoSorb hemadsorption treatment, 9 studies reported COVID-19 patients with ECMO support, with a pooled incidence of ECMO support of 73.2% (95% CI 48.7–97.7%).

### Mortality for patients with or without ECMO

There are eight studies reported the in-hospital mortality for patients received CytoSorb therapy with ECMO support. The in-hospital mortality reported varied from 27.3%-82.4%, and the pooled in-hospital mortality of COVID-19 patients receiving CytoSorb therapy with ECMO support was 54% (95% CI 36.9%-71.1%, I^2 =^ 74%) (**Supplementary Figure 4**). For patients without ECMO support, it was reported in eight studies, the mortality varied from 18.2%-76.9%, and the pooled in-hospital mortality of COVID-19 patients receiving CytoSorb therapy without ECMO support was 37.6% (95% CI 21.2%-54.1%, I^2 =^ 70%) (**Supplementary Figure 5**).

## Discussion

This prompt review of the relevant evidence and subsequent meta-analysis studied the efficiency of CytoSorb hemadsorption in adult COVID-19 patients. The pooled in-hospital mortality in these COVID-19 patients was 42.1%, and this estimate was supported by highly credible evidence. However, a study reported in-hospital mortality of 37% for COVID-19 patients with ECMO support (33). This implies that mortality was slightly higher (42.1%) in patients receiving CytoSorb hemadsorption treatment. The pooled mean serum IL-6 and CRP were 168.83 and 92.41 mg/L, respectively. The values indicate that compared with the baseline levels before the application of CytoSorb, both IL-6 and CRP levels have decreased after hemadsorption treatment. The pooled mean duration of MV was 14.37 days and the pooled mean length of ICU stay was 19.74 days. The majority of patients (73.2%) received adjunctive ECMO support.

COVID-19 triggers cytokine storm, a systemic inflammatory reaction that increases immune cell activation and pro-inflammatory cytokine production (34). Furthermore, a postmortem examination of the lung of a person who died of COVID-19 revealed the presence of acute respiratory distress syndrome (ARDS) and T-cell overactivity (35). Consequent to infection with SARS-CoV-2, innate and adaptive immune responses were triggered, resulting in uncontrolled inflammatory responses and, eventually, the cytokine storm (36). Several studies found that seriously sick COVID-19 patients had greater levels of pro-inflammatory cytokines, particularly IL-6, than moderately unwell individuals (37, 38). The increase of IL-6 and IL-10 in the COVID-19 patients was constant. IL-6 targets the IL-6 receptor, and the latter recruits JAK, which activates the signal transducer and activator of transcription 3 through a cascade signal (39). The transcriptome sequencing of bronchoalveolar lavage fluid (BALF) cells indicated that SARS-CoV-2 infection causes increased chemokine releases, such as C-X-C motif chemokine ligand 10 (CXCL-10) and chemokine C-C motif ligand 2 (CCL-2) (40). The excessive release of cytokines in COVID-19 patients also suggests a bad prognosis. The cytokine storm may cause epithelial and endothelial cell apoptosis and vascular leakage, which can lead to ARDS, other severe symptoms, and eventually to death.

Serum IL-6 concentrations can be reduced from a median value of 5000 to 289 pg/mL after 24 h of cytokine adsorption in patients with severe infections (41). IL-6 levels have been associated with increased severity and mortality in COVID-19 patients. Herein, the pooled results indicated that the mean serum IL-6 level decreased from 339.49 to 168.83 pg/mL. The RECOVERY trial showed benefits in COVID-19 patients with low-dose dexamethasone (42). Although CytoSorb treatment demonstrated effective suppression of inflammation in COVID-19 patients, the in-hospital mortality remains higher (42.1%) compared with ECMO-support patients (36.9%) (43). One possible explanation could be these patients were severely ill with high SOFA and APACHE2 scores.

Although CytoSorb device has neither been cleared nor approved by the Food and Drug Administration (FDA) for the indication of treating patients with COVID-19. In April 2020, CytoSorb therapy was granted by Emergency Use Authorizations (EUA) to adsorb inflammatory cytokines in adult COVID-19 patients admitted to the ICU with imminent or confirmed respiratory failure (44). Therefore, early acute lung injury, early acute respiratory distress syndrome, septic shock, and multiple organ dysfunction are indications to initiate CytoSorb therapy in adult COVID-19 patients. However, exact criteria for hyperinflammation did not defined by EUA, the decision to initiate CytoSorb therapy is at the discretion of the treating doctor. What’s more, The CytoSorb device can be easily integrated into extracorporeal circuits including continuous renal replacement therapy (CRRT) and ECMO. In this way, CytoSorb is also suitable for patients with renal dysfunction or cardiopulmonary failure as adjuvant therapy.

Our study concludes that when conventional treatment fails to provide adequate clinical stability to the patient, CytoSorb therapy should be explored as an additional therapy. Although patients included in our study were critically ill with 73.2% of them being supported with ECMO, the in-hospital mortality was lower. It is to be noted that CRP is a very sensitive marker of inflammation and tissue damage. Our study showed that after CytoSorb treatment, the CRP level decreased. The removal spectrum of CytoSorb seems to encompass other inflammation-related trigger substances such as damage-associated molecular patterns, which could provoke and maintain a generalized inflammatory host response.

Despite certain important insights obtained, we recognize several limitations of the current study. First, the research was primarily a case series study, except for the inclusion of one randomized controlled trial (RCT). Although publication bias was absent and JBI critical appraisal indicated most of the articles as high quality and suitable for inclusion, a relatively small sample size of these studies may induce increased heterogeneity in our results. Including the primary outcome of in-hospital mortality, other pooled results also indicated high heterogeneity. Second, the application of CytoSorb is a new technique that has been widely used in systemic hyperinflammation patients. However, there is no experience in applying this blood purification technique to COVID-19 patients. Hence, any standard protocol for CytoSorb usage is absent to date and the pharmacological treatments and application procedures of CytoSorb may be diverse for different countries and centers. Therefore, these confounding factors inevitably affected outcomes. Third, as the advantages of CytoSorb in COVID-19 patients have not yet been fully investigated, only a small number of studies each with a very small sample size reported Cytosorb treatment for COVID-19 patients. Hence, the credibility of the conclusion from our meta-analysis may not be sufficient. Therefore, more RCTs with larger sample sizes will be needed to validate the conclusion.

In summary, this single-arm proportion meta-analysis demonstrated that the majority of COVID-19 patients treated with CytoSorb also received ECMO support. Furthermore, both CRP and IL-6 levels decreased after Cytosorb treatment, and the pooled in-hospital mortality was 42.1%. However, more RCTs are required to confirm the efficiency of Cytosorb treatment in COVID-19 patients.

## Author contributions

YL proposed the view, designed and participated in the whole research process. Literature searching, screening, and data extracting was carried out by SW, YZ, KZ, JL, and ML. The data was checked and graphs and tables were generated by SW and YZ. The data results were analyzed by JY and RZ. YL and ZL provided guidance on the research and contributed to the final version of the paper. SW and YZ contributed equally to the overall research. A decision was made between YL and ZL regarding the specifics of publication of the article. All authors contributed to the article and approved the submitted version.
