# Supplementary material for: CytoSorb in patients with coronavirus disease 2019: A rapid evidence review and meta-analysis
Source: Front Immunol. 2023 Jan 31;14:1067214. doi: 10.3389/fimmu.2023.1067214 (PMC9927009; doi:10.3389/fimmu.2023.1067214)
Supplement: Supplementary file 1 [file DataSheet_1.pdf]

Supplementary Figures and Tables

Table S1 JBI checklist

Study	1	2	3	4	5	6	7	8	9	10	11	Score
Case series												
Alharthy	✓	✓	✓	✓	✓	✓	✓	✓	✓	✓	-	10
Damiani	✓	✓	✓	✓	✓	✓	✓	✓	✓	✓	-	10
Mehta	✓	✓	✓	✓	✓	✓	✓	✓	✓	✗	-	9
Nassiri	✓	✓	✓	✓	✓	✓	✓	✓	✓	✓	-	10
Paisey	✓	✓	✓	✗	✗	✓	✓	✓	✓	✓	-	8
Peng	✓	✓	✓	✗	✓	✓	✓	✓	✓	✓	-	9
Pieri	✓	✓	✓	✓	✗	✓	✓	✓	✓	✓	-	9
Rampino	✗	✓	✓	✓	✗	✓	✓	✓	✓	✓	-	8
Rodeia	✓	✓	✓	✓	✗	✓	✓	✓	✓	✓	-	9
Cohort Studies												
Lebreton	✓	✓	✓	✗	✗	✓	✓	✓	✓	✓	✓	9
Lewis	✓	✓	✓	✓	✗	✓	✓	✓	✓	✓	✓	10
Supady	✓	✓	✓	✓	✗	✓	✓	✓	✓	✓	✓	10
Song	✓	✓	✓	✓	✗	✓	✓	✓	✓	✓	✓	10

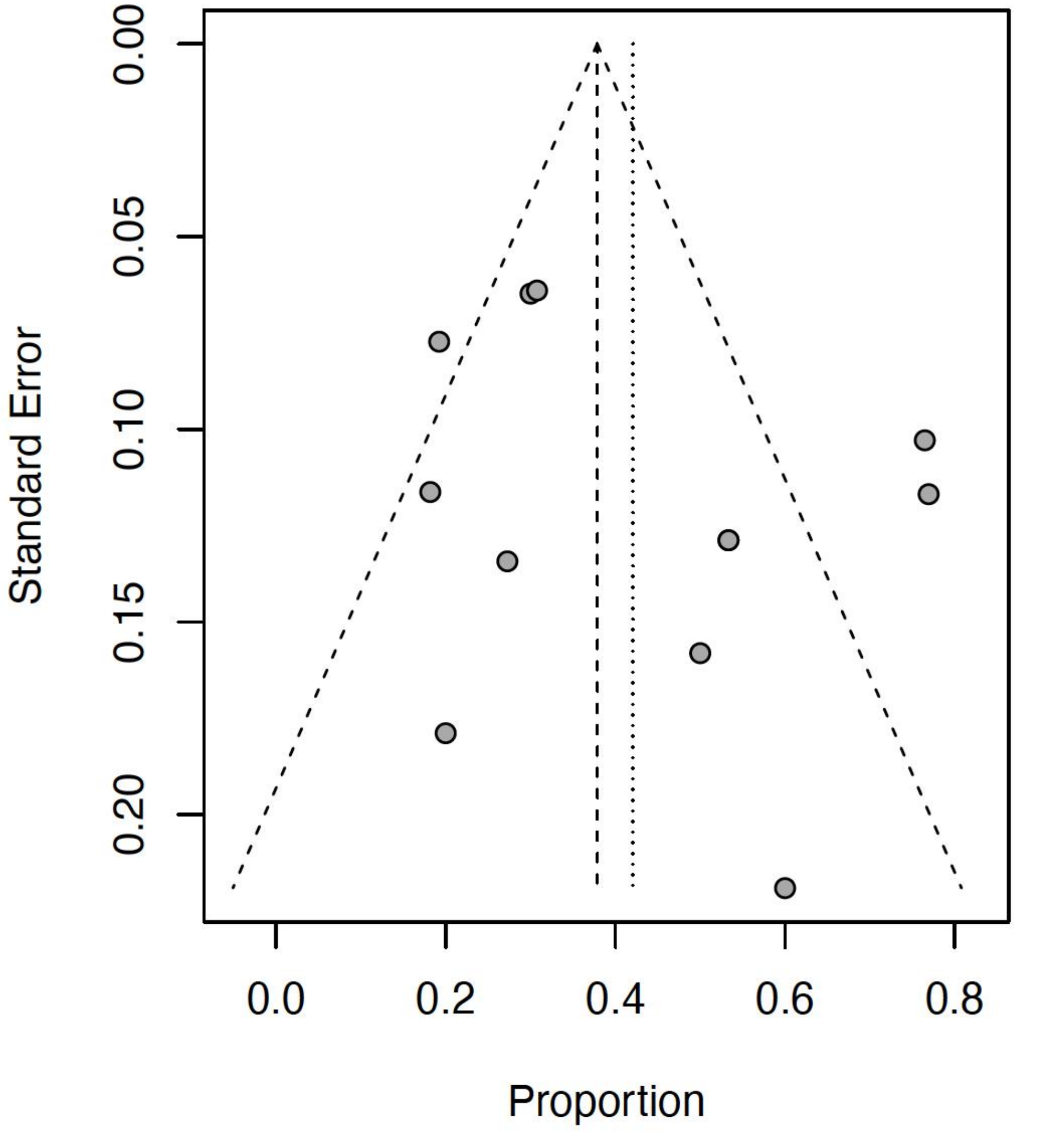

Figure S1: Funnel plot for primary outcome in-hospital mortality

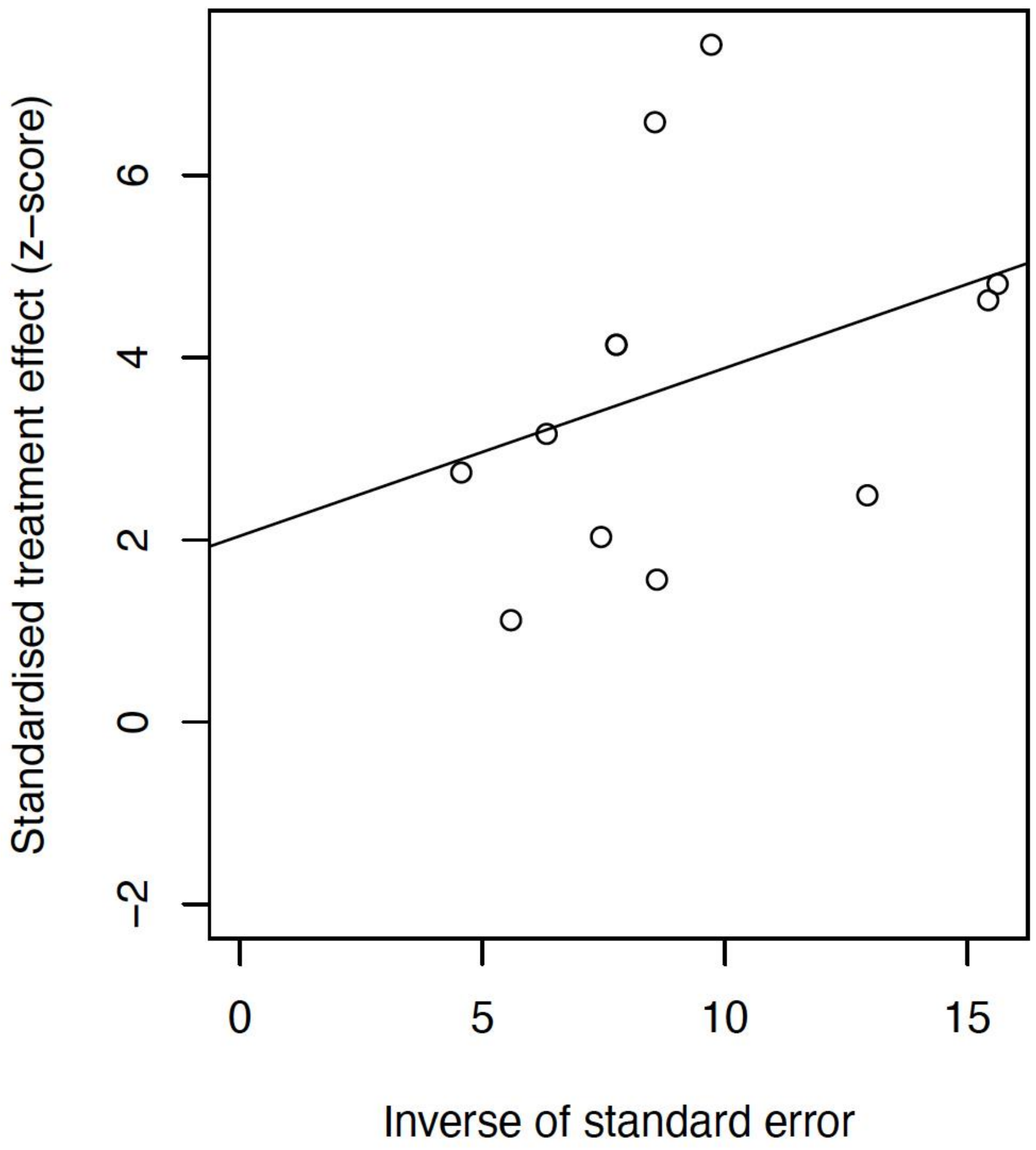

Figure S2: Egger tests to evaluate publication bias

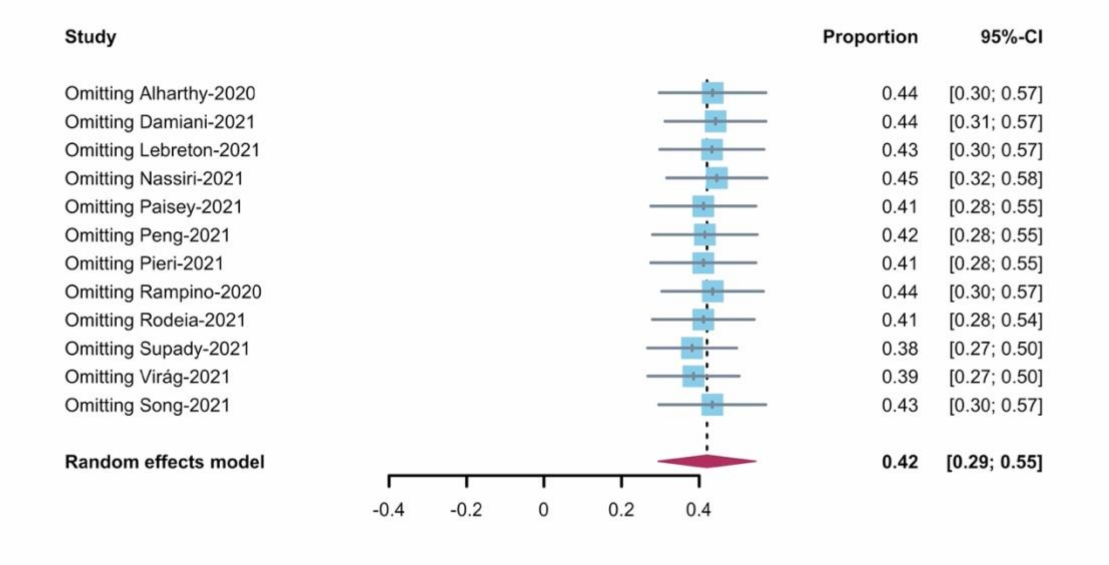

Figure S3: Sensitive analysis for in-hospital mortality using the single-study-removed method

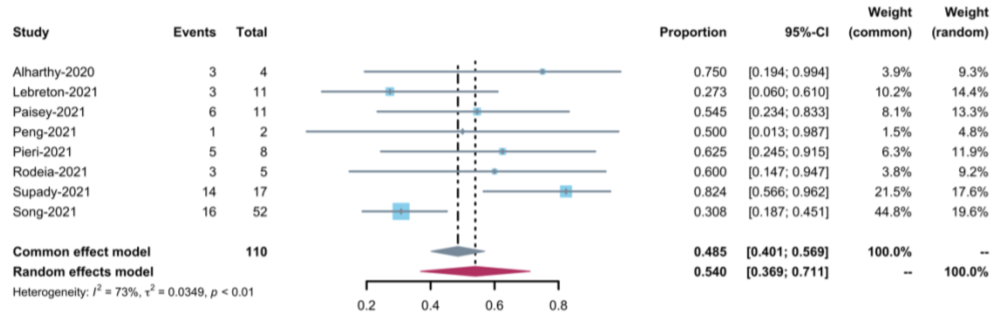

Figure S4: Forest plot of in-hospital mortality for COVID-19 patients who are treated with ECMO. CI: confidence interval.

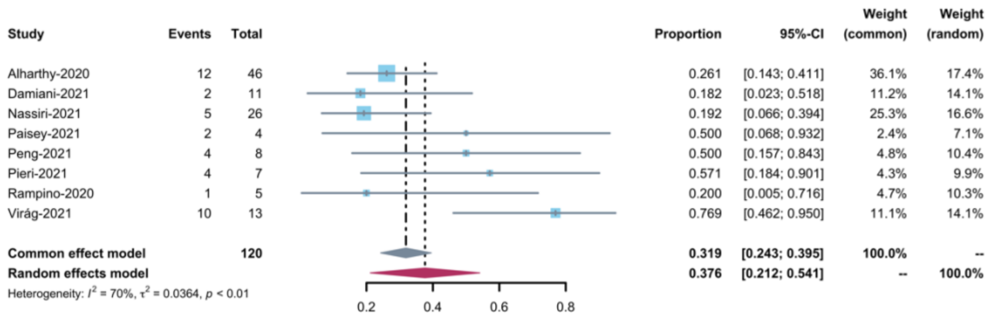

Figure S5: Forest plot of in-hospital mortality for COVID-19 patients who are treated with without ECMO. CI: confidence interval.
